# Tumor regression grade combined with lymph node status in esophageal squamous cell carcinoma after neoadjuvant chemoradiotherapy

**DOI:** 10.1002/cam4.4748

**Published:** 2022-04-17

**Authors:** Jae Kwang Yun, Youngwoong Kim, Geun Dong Lee, Sehoon Choi, Yong‐Hee Kim, Dong Kwan Kim, Seung‐Il Park, Hyeong Ryul Kim

**Affiliations:** ^1^ Department of Thoracic and Cardiovascular Surgery, Asan Medical Center University of Ulsan College of Medicine Seoul Republic of Korea; ^2^ Department of Thoracic and Cardiovascular Surgery Gangnam Severance Hospital, Yonsei University College of Medicine Seoul Republic of Korea

**Keywords:** esophageal cancer, esophageal surgery, neoadjuvant induction therapy, tumor regression grade

## Abstract

**Introduction:**

We aimed to elucidate the prognostic value of tumor regression grade (TRG) combined with lymph node status compared with the 8th edition of the ypTNM staging system in patients with advanced esophageal squamous cell cancer (ESCC) after neoadjuvant chemoradiotherapy (nCRT).

**Methods:**

We enrolled 325 patients with ESCC who underwent nCRT followed by complete resection. We adopted the modified Schneider TRG system, with high (ypT0N0), mid (ypT0N+ or ypT + N0), and low (ypT + N+). After developing a multivariable Cox model, the discrimination ability of the ypStage and TRG systems was evaluated using the Akaike Information Criterion (AIC) and *R* ^2^ measure.

**Results:**

The mean duration of follow‐up was 56.7 ± 43.3 months. The survival curves between the adjacent groups of TRG were significantly different for both overall survival (OS) and recurrence‐free survival (RFS). However, there were no significant differences between ypStages II and III for OS (*p* = 0.683) or RFS (*p* = 0.760). The TRG system also had a discrimination ability in patients with ypStage I (*p* < 0.001 for both OS and RFS) and ypStage III (*p* = 0.045 for OS and 0.042 for RFS). Compared with the ypTNM staging system, the modified TRG had a lower AIC value (1835.99 vs. 1852.02) and a higher *R* ^2^ (0.256 vs. 0.177), indicating better discrimination ability and prediction accuracy.

**Conclusions:**

For patients with ESCC who underwent esophagectomy following nCRT, the modified Schneider TRG system may complement the ypStage and help clinicians select the most appropriate postoperative treatment and surveillance.

## Graphical abstract

The modified Schneider tumor regression grade system showed better discrimination ability and prediction accuracy than the 8th edition of ypStage system. For advanced esophageal cancer, this system may help clinicians select the most appropriate postoperative treatment and surveillance.

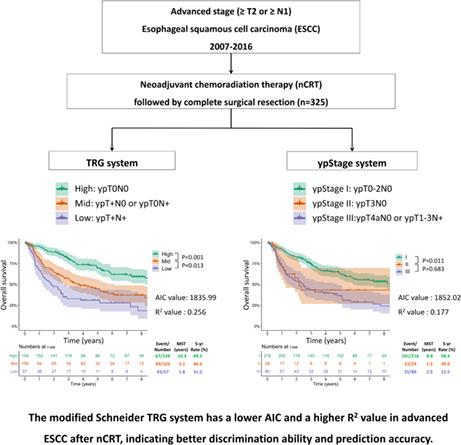

## 1 INTRODUCTION

Locally advanced esophageal cancer (≥T2 or node‐positive disease) has a significantly worse prognosis than early esophageal cancer, with a 5‐year survival rate ranging from 16% to 33%.^1^ According to the National Comprehensive Cancer Network (NCCN) guidelines,^2^ neoadjuvant chemoradiation therapy (nCRT) followed by surgery is the standard treatment for locally advanced esophageal cancer.

Accurate restaging of patients after nCRT is crucial because it offers prognostic information, which can help clinicians make decisions about proper treatment and surveillance. The most frequently used method is the ypTNM staging system updated by the American Joint Committee on Cancer (AJCC).^3^ However, several studies have raised questions about whether this staging accurately reflects the prognosis of the patient after nCRT.^4^, ^5^ Another method is to quantify the cancer response to nCRT, the so‐called tumor regression grade (TRG).

Many previous studies have proposed various score systems to assess pathological response as a way to predict the long‐term prognosis of patients with advanced esophageal cancer (Table 1).^6^, ^7^, ^8^, ^9^, ^10^ Nonetheless, the TRG has never been integrated into a defined staging system for esophageal cancer.^3^ One of the reasons for this is that most TRG systems evaluate only the primary tumor without consideration of regional lymph node status,^6^, ^7^, ^8^, ^9^ which is thought to be a major determinant of prognosis.^11^, ^12^ Moreover, no studies have compared TRG with the ypTNM staging system as a prediction model in locally advanced esophageal cancer.

**TABLE 1:** Tumor regression grade (TRG) systems

Mandard^6^	Chirieac^7^	JES*^9^	Schneider^10^	Modified Schneider^a^
Grade 1: No residual cancer Grade 2: Rare residual cancer cells scattered through the fibrosis Grade 3: Increased residual cancer cells with predominated fibrosis Grade 4: residual cancer with predominant fibrosis Grade 5: no regressive change	Grade 1: No residual cancer Grade 2: 1–50% residual cancer cells Grade 3: No response, >50% residual cancer cells	Grade 1a: Viable cancer cells accounting for 2/3 or more tumor tissue Grade 1b: Viable cancer cells accounting for 1/3 or more, but less than 2/3 of tumor tissue Grade 2: Viable cancer cells account for less than 1/3 of tumor tissue, while other cancer cells are severely degenerated or necrotic Grade 3: No viable cancer cells are evident	Grade 1: Residual cancer cells>1%, with lymph node metastasis (ypN+)|| Grade 2: Residual cancer cells>1%, without lymph node metastasis (ypN0)? Grade 3: Residual cancer cells<1%, with lymph node metastasis (ypN+)|| Grade 4: Residual cancer cells<1%, without lymph node metastasis (ypN0)?	Low: ypT + N+ Mid: ypT + N0 or ypT0N+ High: ypT0N0

Abbreviations: JES*, Japanese Esophageal Society.; ypN0?, no regional lymph node metastases after neoadjuvant therapy; ypN1||: the presence of regional lymph node metastases after neoadjuvant therapy.

^a^ Schneider TRG was modified into 3 categories, namely low (grade 1), mid (grade 2 and 3), and high (grade 4), according to our survival analysis.

In this study, we evaluated the prognostic value of TRG combined with lymph node status for patients with advanced‐stage esophageal squamous cell cancer (ESCC) who underwent nCRT followed by complete surgical resection. In addition, we sought to compare the predictive ability of TRG and ypTNM based on the 8th edition of the AJCC classification.

## 2 METHODS

All clinical records of patients who underwent surgery for esophageal cancer were retrospectively reviewed between January 2007 and December 2017 in the Thoracic Surgery Department of the Asan Medical Center, Seoul, South Korea (*n* = 1123). Exclusion criteria were as follows: (I) histology other than ESCC (*n* = 45); (II) Cervical or abdominal esophageal cancer (*n* = 21); (III) early esophageal cancer without nCRT (*n* = 667); (IV) transhiatal esophagectomy (*n* = 38), and (V) incomplete resection (*n* = 27). This study was approved by the Institutional Review Board of the Ethics Committee of the Asan Medical Center (IRB number: 2018–0557).

Patient workup for pre‐operative staging was performed according to the NCCN guideline, the details of which have been previously described elsewhere.^13^ All patients were administered XP regimen (capecitabine 1600 mg/m^2^ per day for 5 days plus cisplatin 30 mg/m^2^ per day on the first day, weekly), FP regimen (cisplatin 60 mg/m^2^ per day on the first day plus 5‐fluorouracil 1000 mg/m^2^ per day on the second day for 4 days, every 3 weeks), or oxaliplatin/TS‐1 regimen (TS‐1 50 mg orally twice a day daily during the whole period of concurrent chemoradiation therapy, and oxaliplatin 130 mg/m^2^ as an intravenous infusion on day 1, repeated every 3 weeks) as nCRT. The median dose of radiation therapy was 46 Gy (range, 38 to 50.4 Gy), and the fraction size was either 180 cGy or 200 cGy. Since the aim of radiotherapy was neoadjuvant treatment, elective nodal irradiation was used in most cases, and the range of field was customized according to the locations of the primary lesion and metastatic lymph nodes.

Endobronchial ultrasound‐guided fine‐needle aspiration or EUS was used to confirm lymph node metastasis suspected by the imaging modalities. After staging, nCRT was generally performed in patients with ≥T2 or ≥N1, except when the patient was in poor physical condition, or with simultaneous gastric cancer according to the judgment from a multidisciplinary oncology clinic. During the surgery, 3‐field lymph node dissection was selectively performed if the patient had cervical esophageal cancer, advanced T stage upper thoracic esophageal cancer, or a suspected cervical/highest‐ mediastinal lymph node metastasis before nCRT.

**FIGURE 1:**
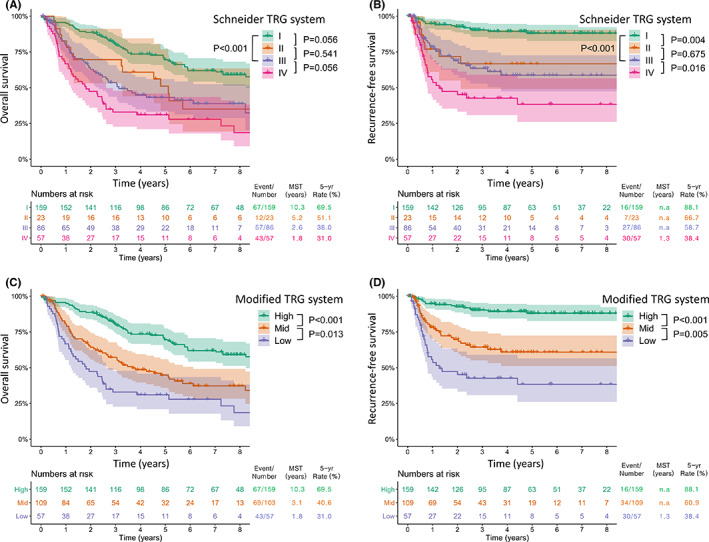
Survival curves for overall survival (A) and recurrence‐free survival (B) by the Schneider tumor regression grade (TRG) system. Survival curves for overall survival (C) and recurrence‐free survival (D) by the modified TRG system

The TRG was classified into 4 categories following the Schneider grading system, which combines the primary tumor and lymph node status together.^10^ TRG grade 1 was defined as residual cancer cells <1% without lymph node metastasis (ypT0N0); grade 2 residual cancer cells <1% with lymph node metastasis (ypT0N+); grade 3 residual cancer cells >1% without lymph node metastasis (ypT + N0); and grade 4 residual cancer cells >1% with lymph node metastasis (ypT + N+). We also used the 3 categories of the modified Schneider grading system, namely high (grade 1), mid (grade 2 and 3), and low (grade 4), according to our survival analysis (Figure 1). Pathologic staging was retrospectively performed according to the 8th edition of AJCC.^3^

If conventional minimal invasive surgery or robot‐assisted surgery was performed without rib resection, we defined it as minimally invasive surgery. We defined in‐hospital death as death within 30 days after surgery or death occurring during the hospital stay without discharge after surgery.

### 2.1 Statistical analysis

Continuous variables are presented as means and standard deviations, and categorical variables as counts and percentages. A parametric test (ANOVA) was used for continuous variables and the chi‐square test was used for categorical variables for comparisons among the three groups. The overall survival (OS) and recurrence‐free survival (RFS) outcomes were compared using Kaplan–Meier curves. The differences in the survival rates were analyzed using the log‐rank test. In case of multiple comparisons (≥3 survival curves), Bonferroni correction was used to calculate the *p* values with the log‐rank test. Cox proportional hazard models were applied to identify the independent factors of OS and RFS. The Akaike Information Criterion (AIC)^14^ and the *R* ^2^ measure^15^ were adopted to calculate the discriminatory values of the final multivariate models based on the TRG and ypTNM staging systems.

All statistical calculations were conducted using R version 3.6.1 (The R Foundation for Statistical Computing) using the “Survival,” “GGally,” “ggplot2,” “Zelig,” “survminer,” and “rms” packages. A *p*‐value of less than 0.05 was considered significant.

## 3 RESULTS

### 3.1 Basal characteristics and surgical profiles

A total of 325 patients who received nCRT followed by complete surgical resection for ESCC were enrolled in this study. Table 2 shows the baseline characteristics of the enrolled patients. The mean duration of follow‐up was 56.7 ± 43.3 months. The distribution of clinical TNM stage was 14.8% (*n* = 48), 43.1% (*n* = 140), and 42.2% (*n* = 137) for stages I, II, and III, respectively. The modified Schneider TRG was not significantly associated with baseline characteristics, except for smoking history (*p* = 0.028).

**TABLE 2:** Baseline characteristics of the study patients

Variables	Total (*n* = 325)	Tumor regression grade			*p* value
		High (*n* = 159)	Mid (*n* = 109)	Low (*n* = 57)	
Age, years	60.4 ± 8.4	60.3 ± 8.4	60.8 ± 8.4	59.6 ± 8.6	0.695
Sex					0.195
Male	302 (92.9)	144 (90.6)	105 (96.3)	53 (93.0)	
Female	23 (7.1)	15 (9.4)	4 (3.7)	4 (7.0)	
BMI*, kg/m^2^	22.8 ± 3.3	23.2 ± 3.4	22.7 ± 3.1	22.2 ± 3.1	0.111
Smoking	266 (81.8)	121 (76.1)	94 (86.2)	51 (89.5)	0.028
Heavy alcohol use	284 (87.4)	133 (83.6)	99 (90.8)	52 (91.2)	0.139
Hypertension	106 (32.6)	55 (34.6)	34 (31.2)	17 (29.8)	0.746
Diabetes mellitus	55 (16.9)	28 (17.6)	14 (12.8)	13 (22.8)	0.253
COPD?	2 (0.6)	0	1 (0.9)	1 (1.8)	0.308
Pulmonary Tbc||	27 (8.3)	10 (6.3)	11 (10.1)	6 (10.5)	0.433
Liver cirrhosis	8 (2.5)	5 (3.1)	3 (2.8)	0	0.409
Arrhythmia	5 (1.5)	3 (1.9)	1 (0.9)	1 (1.8)	0.810
History of stroke	13 (4.0)	7 (4.4)	4 (3.7)	2 (3.5)	0.935
Coronary vessel disease	9 (2.8)	5 (3.1)	2 (1.8)	2 (3.5)	0.759
Tumor location					0.105
Upper (UI# 20–25 cm)	64 (19.7)	15 (9.4)	12 (11.0)	9 (15.8)	
Middle (UI# 26–30 cm)	136 (41.8)	42 (26.4)	32 (29.4)	11 (19.3)	
Lower (UI# 31–40 cm)	125 (38.5)	34 (21.4)	18 (16.5)	17 (29.8)	
Tumor length	4.9 ± 2.9	4.6 ± 3.0	5.2 ± 3.0	5.2 ± 2.5	0.199
Grade of tumor differentiation					0.470
Well	46 (14.2)	27 (17.0)	14 (12.8)	5 (8.8)	
Moderate	139 (42.8)	51 (32.1)	42 (38.5)	22 (38.6)	
Poor	140 (43.1)	70 (44.0)	44 (40.4)	26 (45.6)	
Clinical T category					0.351
cT1	47 (14.5)	28 (17.6)	12 (11.0)	7 (12.3)	
cT2	93 (28.6)	49 (30.8)	28 (25.7)	16 (28.1)	
cT3	185 (56.9)	82 (51.6)	69 (63.3)	34 (59.6)	
Clinical N category					0.068
cN0	90 (27.7)	54 (34.0)	27 (24.8)	9 (15.8)	
cN1	222 (68.3)	101 (63.5)	76 (69.7)	45 (78.9)	
cN2	13 (4.0)	4 (2.5)	6 (5.5)	3 (5.3)	
Clinical TNM stage					0.110
I	48 (14.8)	28 (17.6)	13 (11.9)	7 (12.3)	
II	140 (43.1)	76 (47.8)	42 (38.5)	22 (38.6)	
III	137 (42.2)	55 (34.6)	54 (49.5)	28 (49.1)	

*Note*: Values are numbers (%), or mean ± standard deviation, unless otherwise indicated. Tumor regression grade was classified into 3 categories, namely high (Schneider grade 1), mid (Schneider grade 2 and 3), and low (Schneider grade 4), according to our survival analysis.

Abbreviations: BMI*, body mass index; COPD?, chronic obstructive pulmonary disease; pulmonary Tbc||, pulmonary tuberculosis; UI#, upper incisor.

Table 3 presents the perioperative profiles perioperative clinical outcomes. A pathological complete response (pCR) of the primary tumor (ypT0) was achieved in 182 patients (56.0%), but 23 of them (12.6%) had lymph node metastasis (ypT0N+). The distribution of ypStage was 66.5% (*n* = 216), 8.9% (*n* = 29), and 24.6% (*n* = 80) in stages I, II, and III, respectively. The modified Schneider TRG had an association with ypT status (*p <* 0.001), ypN status (*p* < 0.001), and ypStage (*p* < 0.001). In‐hospital death (*p* = 0.962) and postoperative death within 30 days (*p* = 0.773), 60 days (*p* = 0.481), and 90 days (*p* = 0.571) were similar among the TRG groups.

**TABLE 3:** Perioperative profiles of the study patients according to tumor regression grade

Variables	Total (*n* = 325)	Tumor regression grade			*p* value
		High (*n* = 159)	Mid (*n* = 109)	Low (*n* = 57)	
Surgical approach					0.121
Conventional	281 (86.5)	132 (83.0)	100 (91.7)	49 (86.0)	
Minimally invasive	44 (13.5)	27 (17.0)	9 (8.3)	8 (14.0)	
Anastomosis site					0.230
Transthoracic	209 (64.3)	98 (61.6)	77 (70.6)	34 (59.6)	
Cervical	116 (35.7)	61 (38.4)	77 (70.6)	34 (59.6)	
Type of conduit					0.257
Stomach	312 (96.0)	154 (96.9)	102 (93.6)	56 (98.2)	
Colon	13 (4.0)	5 (3.1)	7 (6.4)	1 (1.8)	
Harvested lymph nodes	34.1 ± 13.7	34.2 ± 14.1	36.3 ± 15.4	36.3 ± 15.4	0.301
ypT status					<0.001
ypT0	182 (56.0)	159 (100.0)	23 (21.1)	0	
ypT1	51 (15.7)	0	32 (29.4)	19 (33.3)	
ypT2	37 (11.4)	0	25 (22.9)	12 (21.1)	
ypT3	55 (16.9)	0	29 (26.6)	26 (45.6)	
ypN status					<0.001
ypN0	245 (75.4)	159 (100.0)	86 (78.9)	0	
ypN1	72 (22.2)	0	23 (21.1)	49 (86.0)	
ypN2	8 (2.5)	0	0	8 (14.0)	
ypStage					<0.001
I	216 (66.5)	159 (100.0)	57 (52.3)	0	
II	29 (8.9)	0	29 (26.6)	0	
III	80 (24.6)	0	23 (21.1)	57 (100.0)	
Early mortality					
In‐hospital death	10 (3.1)	5 (3.1)	3 (2.8)	2 (3.5)	0.962
Within 30 days	2 (0.6)	1 (0.6)	1 (0.9)	0	0.773
Within 60 days	6 (1.8)	4 (2.5)	2 (1.8)	0	0.481
Within 90 days	10 (3.1)	4 (2.5)	3 (2.8)	3 (5.3)	0.571

*Note*: Values are numbers (%) or mean ± standard deviation, unless otherwise indicated. Tumor regression grade was classified into 3 categories, namely high (Schneider grade 1), mid (Schneider grade 2 and 3), and low (Schneider grade 4), according to our survival analysis.

### 3.2 Survival analysis

**FIGURE 2:**
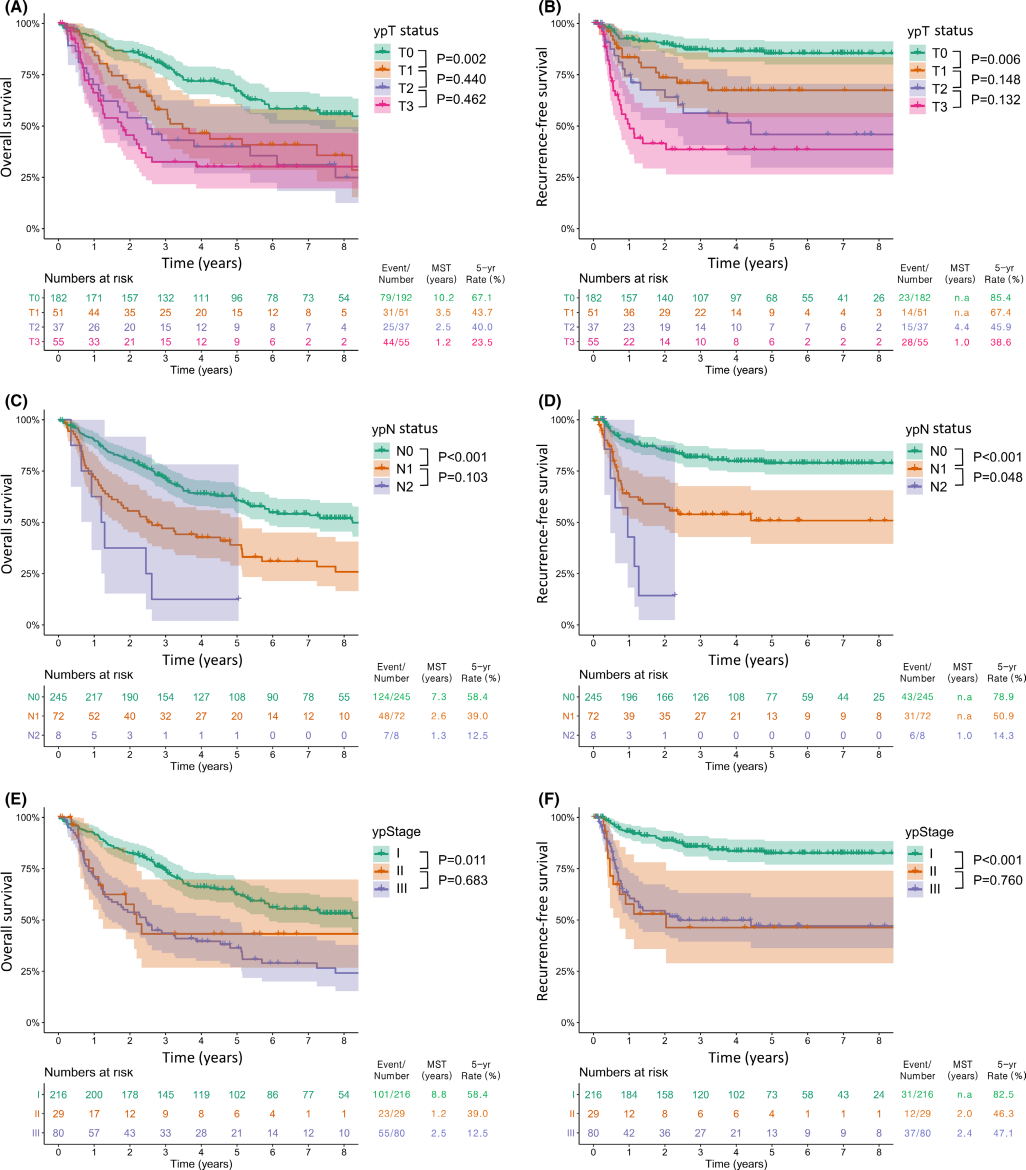
Survival curves for overall survival (A) and recurrence‐free survival (B) by the ypT status. Survival curves for overall survival (C) and recurrence‐free survival (D) by the ypN status. Survival curves for overall survival (E) and recurrence‐free survival (F) by the ypStage

**FIGURE 3:**
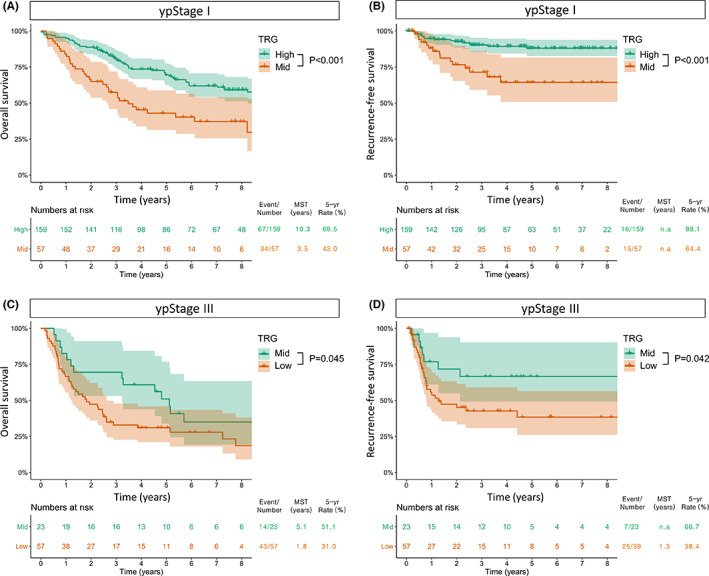
Survival curves for overall survival (A) and recurrence‐free survival (B) by the modified TRG system in ypStage I. Survival curves for overall survival (C) and recurrence‐free survival (D) by the modified TRG system in ypStage III

The Kaplan–Meier survival curves according to TRG are plotted in Figure 1. For the 4 categories of the Schneider TRG system, the 5‐year OS rates were 69.5%, 51.1%, 38.0%, and 31.0% for grades I, II, III, and IV, respectively (Figure 1A). With regard to RFS, they were 88.1%, 66.7%, 58.7%, and 38.4% for grades I, II, III, and IV, respectively (Figure 1B). After combining the Schneider grades II and III, which had similar survival outcomes, into a single group (3 categories), we could obtain statistically significant differences between each group, irrespective of OS and RFS (Figure 1C,D). As for the ypT (Figure 2A,B) and ypN (Figure 2C,D) status, a phased degradation was shown between each group for both OS and RFS, albeit without statistical significance. However, in ypStage, the curves of stage II and III were overlapping (*p* = 0.683 for OS and 760 for RFS) (Figure 2E,F). Subgroup analyses were performed according to TRG in patients by individual ypStage (Figure 3). As a result, TRG still had a discrimination ability in patients with ypStage I (*p* < 0.001 for both OS and RFS) (Figure 3A,B) and ypStage III (*p* = 0.045 for OS and 0.042 for RFS) (Figure 3C,D).

Table 4 summarizes the results of the multivariable Cox analysis based on the modified TRG (model 1) and ypTNM staging system (model 2). For the TRG‐based model, prognostic factors for OS were tumor length, conduit type, tumor differentiation grade, and TRG, whereas it was only TRG for RFS (Table 4, model 1). Except for the ypStage instead of the TRG system, the other variables were the same in the ypStage‐based model. Compared to the ypStage‐based model, TRG had a better model fit as indicated by the smaller AIC values (OS: 1835.99 vs. 1852.02, RFS: 828.14 vs. 837.63) and better prediction accuracy as indicated by the higher R^2^ values (OS: 0.256 vs. 0.177, RFS: 0.493 vs. 0.430) for both OS and RFS.

**TABLE 4:** Multivariable analysis of prognostic factors for patients with advanced esophageal cancer who received nCRT based on TRG (model 1) and overall ypStage (model 2)

	Overall survival		Recurrence‐free survival	
	*p* value	HR (95% CI)	*p* value	HR (95% CI)
Model 1 (TRG‐based)				
Tumor length	0.024	1.06 (1.01–1.11)		
Type of conduit (vs. stomach)				
Colon	0.012	2.36 (1.21–4.61)		
Tumor differentiation grade (vs. well)				
Moderate	0.026	2.80 (1.13–6.95)		
Poor	0.013	2.90 (1.25–6.73)		
Tumor regression grade (vs. high)				
Mid	<0.001	2.20 (1.56–3.10)	<0.001	4.15 (2.29–7.53)
Low	<0.001	3.28 (2.22–4.85)	<0.001	8.39 (4.56–15.45)
AIC for model 1	1835.99		828.14	
*R* ^2^ for model 1	0.256		0.493	
Model 2 (ypStage‐based)				
Tumor length	0.022	1.06 (1.01–1.11)		
Type of conduit (vs. stomach)				
Colon	0.013	0.34 (1.20–4.56)		
Tumor differentiation grade (vs. well)				
Moderate	0.033	2.70 (1.08–6.74)		
Poor	0.019	2.75 (1.19–6.39)		
ypStage (vs. ypStage I)				
II	0.029	1.88 (1.07–3.30)	<0.001	5.05 (2.59–9.87)
III	<0.001	2.20 (1.58–3.05)	<0.001	4.49 (2.78–7.25)
AIC for model 2	1852.02		837.63	
*R* ^2^ for model 2	0.177		0.430	

Abbreviations: AIC, Akaike information criterion; CI, confidence interval; HR, hazard ratio; nCRT, neoadjuvant chemoradiation therapy; TRG, tumor regression grade.

## 4 DISCUSSION

We demonstrated the prognostic value of TRG in ESCC after nCRT followed by surgery. Using the modified Schneider TRG system that simultaneously reflects the primary tumor status and lymph node metastasis, we found a statistically significant difference in the survival outcome between the groups, which was not seen in the ypTNM staging system. Given the results of AIC and R^2^ values, the discrimination ability of TRG was higher than the ypTNM stage, irrespective of OS and RFS.

Numerous studies have suggested different grading systems for TRG in esophageal cancer.^6^, ^7^, ^8^, ^9^, ^10^ The most widely used TRG systems are those of Mandard et al.,^6^ Chirieac et al.,^7^ and the Japanese Esophageal Society system^9^ (Table 1). However, these systems have an important problem of using ambiguous terms for their definitions. For example, “rare” residual cancer cells are defined in Mandard TRG 2 or “predominant” fibrosis in Mandard TRG 3, which results in interobserver variability.^6^ Cutoff values used in the current TRG systems (e.g., >50% or 1/3 to 2/3) might also be a factor that increases variability among observers.^7^, ^9^ Consequently, Chetty et al. reported that the concordance rate was very low among experienced gastrointestinal pathologists, even when using the same TRG system.^16^

Another critical problem is that the current TRG systems do not consider the ypN status. ypN status has been reported to be the major determinant of prognosis,^11^, ^12^ and a pCR is technically ypT0N0.^17^ However, current TRG systems do not distinguish ypT0N0 and ypT0N+, which have been proven to have different prognoses in Lv et al.’s report^12^ and also in our study (Figure 1). Considering the points described above, we selected the modified Schneider grading system,^10^ which simultaneously incorporates the primary tumor and lymph node status. Moreover, to reduce the interobserver variability by the cutoff value, we regarded the definition of “residual cancer cells <1%” as no residual cancer (ypT0).

The introduction of the ypTNM was one of the major modifications in the 8th edition of the AJCC classification.^3^ Although the ypStage showed a slightly better predictive accuracy than the pStage,^18^ the prognostic value of the ypTNM system has often been challenged.^4^, ^5^ First of all, ypT status shares the same categories with pT status that were originally targeted for treatment‐naïve tumors. However, it is difficult to measure the exact depth of the primary tumor after nCRT in the real world. Furthermore, there is no evidence that tumor regression proceeds from the deepest to the superficial layer after nCRT. Various conditions could be included as ypT3 status from minimal residual cancers in the adventitia (with complete clearance of tumor cells in the mucosa, submucosa, and muscle layers) to near‐native cancers without response to nCRT. In this scenario, the incorporation of TRG into ypT staging may offer superior prognostic stratification, especially in patients with advanced ypT status. Second, the AJCC system does not adequately emphasize the clinical significance of a pCR (ypT0N0), which has a distinctly favorable prognosis. According to our study, in patients with ypStage I (ypT0‐2 N0), the high TRG group (ypT0N0) had a better prognosis than the mid‐TRG group (ypT1‐2 N0) (Figure 3A,B). In addition, there was a significant prognostic difference between the mid (ypT0N+) and low TRG group (ypT + N+) in patients with ypStage III (Figure 3C,D). We believe these findings are the main reason why our TRG system showed a better discrimination ability than the ypTNM staging system (Table 4).

Consequently, given the modified TRG system, we propose to classify ypT0N0 solely as ypStage I and ypT1‐2 N0 as ypStage II together with ypT3N0. In addition, ypT0N1–2 is recommended to reclassify from ypStage III to ypStage II (Table S1). We think these amendments will increase the survival rates of the patients with current ypStage II, who have a similar prognosis as those with ypStage III (Figure 2E,F), which in turn will improve the overall discrimination ability (Figure 1C,D).

There are several limitations in the current study: (1) this study was retrospective, observational, and nonrandomized, and may therefore be prone to selection or information bias; (2) a relatively small number of patients were enrolled in the current study, thereby several outcomes with marginal significance in our study might have statistical significance in reality. A prospective, international collaborative study is needed to obtain a large independent data set with a sample size sufficient for external validation.

In conclusion, our modified Schneider TRG system, which evaluates both the primary tumor and metastatic lymph nodes, showed a better predictive value for OS and RFS than the 8th edition of the ypTNM staging system. This TRG system may complement ypStage and help clinicians make appropriate decisions about postoperative treatment and surveillance of patients with advanced esophageal cancer who underwent nCRT followed by surgery.

## CONFLICT OF INTEREST

All authors declare there is no financial relationship with a commercial entity that has an interest in the subject of the presented manuscript or any other conflicts of interest to disclose.

## AUTHOR CONTRIBUTION

Designing the study: Hyeong Ryul Kim. Collecting, analyzing, and interpreting the data: Jae Kwang Yun, Youngwoong Kim, Geun Dong Lee, Sehoon Choi, Hyeong Ryul Kim, Seung‐Il Park, Yong‐Hee Kim. Writing the report: Jae Kwang Yun, Youngwoong Kim. Making the decision to submit for publication: Hyeong Ryul Kim.

## Supporting information


Table S1

## Data Availability

The authors confirm that the data supporting the findings of this study are available within the article [and/or] its supplementary materials.
