# Supplementary material for: Tumor regression grade combined with lymph node status in esophageal squamous cell carcinoma after neoadjuvant chemoradiotherapy
Source: Cancer Med. 2022 Apr 17;11(19):3623–32. doi: 10.1002/cam4.4748 (PMC9554450; doi:10.1002/cam4.4748)
Supplement: Supplementary file 1 — Table S1 [file CAM4-11-3623-s001.docx]

Supplementary Table 1. Proposals for revision of the ypStage groupings in the forthcoming (9th) edition of the TNM classification for esophageal cancer

| Modified Schneider# | Proposed 9^th^ ypStage group | ypT | ypN | ypM |
| --- | --- | --- | --- | --- |
| High  (ypT0N0) | I | T0 | N0 | M0 |
| Mid  (ypT+N0 or ypT0N+) | II | T1–3  T0 | N0  N1–2 | M0  M0 |
| Low  (ypT+N+) | III | T1–4  T4 | N1–3  N0 | M0  M0 |
|  | IV | T1–4 | N0–3 | M1 |

#Schneider TRG was modified into 3 categories, namely high (grade 1), mid (grade 2 and 3), and low (grade 4), according to our survival analysis.
